# Nutritional and Psychological Considerations for Dietary Therapy in Eosinophilic Esophagitis

**DOI:** 10.3390/nu14081588

**Published:** 2022-04-12

**Authors:** Javier Molina-Infante

**Affiliations:** 1 Department of Gastroenterology, Hospital Universitario de Caceres, 10004 Caceres, Spain; xavi_molina@hotmail.com; 2 Centro de Investigación Biomédica en Red de Enfermedades Hepáticas y Digestivas (CIBERehd), Instituto de Salud Carlos III, 28029 Madrid, Spain

**Keywords:** eosinophilic esophagitis, diet, nutrition

## Abstract

The step-up empiric elimination diet, starting from one/two food groups of most local allergens remains the current gold standard for a dietary approach in eosinophilic esophagitis (EoE) patients. Milk, followed by wheat and egg, is the most frequent food that triggers EoE in pediatric and adult patients. Elimination diets, with restrictions over four food groups, may be limited to highly motivated patients, in which nutritional counseling is recommended. Malnourishment is uncommon in EoE patients and likely multifactorial (concomitant gastrointestinal eosinophilic disorders or IgE-mediated food allergies, feeding difficulties, abnormal feeding behavior). Avoidant/restrictive food intake disorder in EoE children on highly restrictive diets was lately described and may warrant specific psychological support. As for adults, quality of life may be impaired by symptom severity and dietary restrictions, aside from recently reported food impaction-related specific anxiety in up to 43% of patients. Severe symptoms, feeding dysfunction, and diet restrictions may negatively influence psychosocial adjustment for patients and their caregivers.

## 1. Introduction

Eosinophilic esophagitis (EoE) is an allergic antigen-induced chronic disease, limited to the esophagus, characterized by symptoms of esophageal dysfunction and eosinophil-predominant esophageal inflammation [1]. In 1995, eight pediatric patients with refractory EoE, attributed then to gastrointestinal reflux disease, achieved clinic and histologic remission on an amino acid-based formula devoid of allergenic content (elemental diet) [2]. Since then, EoE is considered a disease mostly triggered by food antigens. Available food allergy testing in clinical practice (IgE-based blood tests against foods, atopy patch tests, skin prick tests) does not accurately predict triggering foods causing EoE in children or adults [1,3]. An elemental diet consists of exclusive feeding by amino acid-based formula. It is the most effective dietary approach, with histologic remission of around 90% for children and adults in a recent meta-analysis [3]. Nonetheless, this approach may not be feasible in routine clinical practice owing to numerous reasons, which include poor palatability (partially improved with recent flavoring), poor psychosocial adjustment, impaired quality of life, and high cost in the case of not being reimbursed. An elemental diet might potentially be used for refractory EoE patients that are willing to be kept in deep remission whilst evaluating the causal role of unusual foods and aeroallergens, or merely as a bridge dietary scheme while investigational drugs are not available. Therefore, empiric elimination diets are currently the gold standard for the dietary management of EoE.

### Step-Up Empiric Elimination Diet: The Current Gold Standard in Dietary Therapy

Back in 2006, a novel empiric diet eliminating six food groups (milk, wheat, egg, soy/legumes, nuts, fish/seafood) that accounted for most food reactions locally, led to complete histologic remission in three out of four pediatric EoE patients from Chicago [4]. After validation of these initial pediatric results in two big studies in adults [5,6], a 6-FED became the standard for dietary therapy in clinical practice. Nevertheless, this dietary approach did not become popular for patients and caregivers due to a need for numerous endoscopies and a high level of restriction for almost a year. Individual food reintroduction in EoE patients achieving histological remission on a 6-FED, each followed by an endoscopic procedure, was instrumental for adequate identification of food triggers in EoE [7]. Cow’s milk (especially in children <10 years old), wheat and egg, and to a lesser extent, soy and legumes, were the most common food triggers for EoE in both children and adults [7]. Therefore, fish/seafood and nuts played a negligible role as causative foods in EoE. 

Upon these findings, a new four-food elimination diet (4-FED) was developed just by eliminating the four more common food groups triggering EoE. A multicenter study conducted in Spanish adult EoE patients (*n* = 52) demonstrated histologic remission in 54% of patients [8]. Half of the responders to a 4-FED had milk, wheat, or both as triggering foods. In a similar fashion, 78 children in a multicenter US study showed an even higher histological remission rate (64%) [9]. Interestingly, milk was the single causative food in up to 55% of pediatric responders to a 4-FED. Therefore, studies on a 4-FED proved that around half of responders had actually one or two food triggers (usually milk and wheat), so they could have accomplished histologic remission by starting with a simpler dietary scheme: a two-food elimination diet (2-FED, withdrawing cow´s milk and wheat). This novelty was first evaluated in 2018 in a big multicenter study gathering 130 consecutive adults and children suffering from EoE, mostly from Spain [10]. All recruited patients underwent a 2-FED (cow´s milk and wheat) and non-responders were progressively escalated to a 4-FED/6-FED if no histological remission (<15 eos/HPF) was documented. A 2-FED led to clinical and histological remission in 43% of patients, whereas a 4-FED (in stepped-up non-responders to a 2-FED) and a 6-FED (in stepped-up non-responders to a 4-FED) achieved similar remission rates (4-FED 60%, 6-FED 79%) to those reported in previous studies [4,5,6,8,9]. When this strategy was compared to a top-down approach (e.g., starting with a 6-FED), the step-up scheme was able to reduce endoscopic procedures and shorten the diagnostic process time by 20%. Moreover, unnecessary dietary restrictions were avoided in over half of the patients, since 43% of patients successfully found their triggering foods without withdrawing egg, legumes, nuts, and fish/seafood, whereas up to 60% went through dietary elimination regularly consuming nuts and fish/seafood. In addition, 80%–90% of responders to a 2-FED or 4-FED were found to have just one or two causative food groups. Responders to simple dietary restrictions with few causative foods, early identified without requiring a 6-FED, may be the best candidates for long-term dietary therapy. As such, a step-up empirical elimination diet, starting from one or two food groups, remains the current gold standard for a dietary approach in children and adult patients with EoE. In a further computer-based simulation model, a 1–3 and 2–4 step-up approach were found to be easier and more efficient strategies [11]. Importantly, a prospective multicenter study evaluating the efficacy of a single food milk elimination diet for EoE children was recently first published [12]. This dietary restriction kept histologic remission in half of the patients, albeit up to 88% were also on PPI therapy (despite the fact of being non-responders to PPIs, according to current guidelines) [12]. Co-therapy with PPIs casts doubt on whether partial responders to PPIs were included or synergistic effects between diet and PPIs were responsible for their notable efficacy. In any case, this study opens up the possibility of even simpler initial dietary approaches in children.

Unlike patients achieving remission on a 2-FED or a 4-FED, those who were escalated to a 6-FED with successful histologic response were found to have from three to six different causative food triggers [10]. Hence, the more we escalate in the step-up dietary strategy, the higher the likelihood of having more food triggers. Long-term and durable histologic response with empiric elimination diets is related to strict adherence to the avoidance of triggering foods [6,13,14]. The avoidance of triggering foods may be troublesome in the long run, even unfeasible with multiple triggering foods, resulting in non-adherence in up to 70% of responders to a 6-FED [15]. Long-term adherence to a 6-FED was significantly influenced by therapy efficacy, social limitations, and diet-related anxiety [16]. Accordingly, a 6-FED may not be generally recommended after failure of a 4-FED, whereas individualized indications might be discussed for patients unwilling to take drugs and/or are still eager to decipher individual causative foods, despite being numerous. All available empiric elimination diets with data on efficacy, food triggers, and potential drawbacks are summarized in Table 1.

## 2. Nutritional Considerations

Elimination diets alone do not represent a major risk for impaired nutritional status, since only foods identified as triggers after food challenge and subsequent endoscopy are withdrawn in the long-term [17]. Most patients will have one or two food triggers when using a step-up approach, therefore ensuring a safety profile for current dietary therapy [10].

### 2.1. Weight and Growth

A recent systematic review revealed that most EoE adult patients exhibit a good nutritional status and expected body mass index (BMI) values when compared to healthy controls [18]. As for children, two recent studies from the US have consistently reported that EoE pediatric patients show minimal baseline impairment of height, while they usually achieve their expected growth, regardless of treatment modality, including elimination dietary therapy [19,20]. 

### 2.2. Failure to Thrive and Malnourishment

While adults with EoE do not usually show malnourishment, conflicting results were reported in the few studies published so far addressing the nutritional status of EoE pediatric patients [18,19,20,21,22,23,24,25]. Failure to thrive/malnutrition were reported in a wide spectrum including 10% [21], 24% [22], or even 31% [23] in EoE pediatric patients. Discrepancies among studies might be explained by selection bias in a majority of retrospective studies (31% in a cohort of children in which 29% had neurological/developmental disorder and 6% were on a gastrostomy tube [23]) and a varying definition of failure to thrive (24% fulfilling one out of six criteria for failure to thrive [22]). A more recent retrospective study [25] could not find malnourishment in terms of growth of BMI mean values compared to those found in controls. Potential explanations for malnourishment in EoE patients are displayed in Table 2. Concomitant eosinophilic gastrointestinal disorders (EGIDs) may be present in malnourished EoE patients. Weight loss and failure to thrive are common in children suffering from EGIDs [26], with high rates of hypoproteinemia (27%), weight loss, and failure to thrive in non-EoE EGIDs patients, when compared to EoE [27]. Similarly, co-existing IgE-mediated food allergies in EoE were reported to worsen clinical presentation of pediatric EoE, with more severe symptoms and lower appetite, compared to EoE without IgE-mediated food allergies [28]. Recently, an observational study from the UK found that children suffering from IgE- and non-IgE-mediated food allergies that eliminated three or more foods were at the highest risk for lower weight [29]. 

Empiric elimination diets for EoE pose a minimal risk for macronutrient intake imbalance, albeit common sources of fats (milk, egg), protein (milk, egg, legumes), and carbohydrates (wheat) are commonly removed from the diet [34]. In this regard, restrictive dietary interventions alone (e.g., elemental diet, 6-FED) have not been shown to impair nutritional status in pediatric EoE patients [19,24]. This finding stresses the fact that patients suffering from multiple IgE-mediated food allergies, EGID, or severe unrelated comorbidities might not be good candidates for dietary therapy, due to an increased risk of malnutrition.

### 2.3. Vitamin Deficiencies

Several studies have consistently reported normal levels for vitamin D, ferritin, prealbumin, folate, and vitamin B12 in pediatric and adult EoE patients [19,25,34]. In a recent systematic review gathering 137 pediatric patients from five studies (three abstracts), the prevalence of low vitamin D widely varied from 0% to 52% [35]. Therefore, definitive conclusions cannot be drawn until quality evidence is further published.

## 3. Psychosocial Considerations in EoE

### 3.1. Maladaptive Feeding

Disruption of feeding skills during early childhood (which may include sensory, behavioral, oral-motor, communication, and emotional skills), owing to either severe symptoms or highly restrictive diets, can create feeding difficulties [36]. Similarly, avoidance of solid food (e.g., elemental diet) in toddlers or children with known feeding dysfunction may result in impaired oral-motor skills [1,7]. As for symptoms, vomiting and reflux-like symptoms were best described as having a negative impact on EoE children <10 years old [19,30,37]. In a recent study, between a third and half of pediatric EoE patients showed abnormal scores in feeding dysfunction [19]. Clinical features of feeding dysfunction are summarized in Table 3. EoE-associated feeding dysfunction in children may include food refusal, low volume intake, gagging with food, vomiting, preference for liquids over solids, low food variety in diet with poor acceptance of new foods [31]. Parental frustration may be focused on the inability to introduce new foods, vomiting, lengthy mealtimes, and fear of malnourishment [31]. From 10 years old onwards, EoE symptoms change, and dysphagia/food impaction remain as central clinical manifestations [1]. These latter patients may modify their behavior during meals by drinking frequently, chewing thoroughly, and selecting softer foods. These subtle feeding modifications may explain the diagnostic delay in adult patients, which still remains on average 5 to 10 years after symptom onset [1,38]. 

### 3.2. Avoidant/Restrictive Food Intake Disorder

Avoidant/restrictive food intake disorder (ARFID) is a newly classified eating disorder (see diagnostic criteria in Table 4), which has been recently described in two EoE pediatric patients following highly restrictive diets [32]. Both patients who suffered from anxiety were in histological remission, and no other mental or physical comorbidity was diagnosed. ARFID develops from negative eating experiences related to feeding difficulties and esophageal symptoms. Even after an effective treatment that induces EoE remission, negative experiences have become deeply rooted in food avoidance related to medical symptoms, anxiety, and food-related issues [33].

In clinical practice, children with EoE may develop ARFID whilst on restrictive elimination diets, reinforcing the importance of initial minimally restrictive diets (e.g., a step-up strategy [10]) for dietary therapy in EoE. Lately, a cross-sectional study on digestive diseases highlighted that as much as more than half of patients with achalasia, EoE, or celiac disease fulfilled the ARFID diagnostic criteria [33]. As such, the authors questioned whether current diagnostic tools may overestimate ARFID in these diseases. Importantly, food restriction- and food avoidance-related symptoms were related to negative psychosocial outcomes, including depression, anxiety, and decreased social and physical functioning [33].

### 3.3. Quality of Life (QoL) in EoE Patients

In adult patients, recurrent food impaction (with disease and choking anxiety ranking higher) and dietary restrictions were demonstrated to be the most important factors influencing psychosocial domains in health-related QoL [39,40]. Depression in adult patients with EoE has been lately reviewed and rated at around 10% of patients [41]. Of note, 46% of adult patients were recently shown to have esophageal hypervigilance and food impaction-related specific anxiety [42]. Of note, anxiety was the strongest predictor of symptom severity and worsened QoL, outweighing endoscopic and histological markers [42].

Concerning children, the impact of the disease may be much more profound in patients and their caregivers due to concerns with relevant symptoms, feeding dysfunction, dietary restrictions, poor psychosocial adjustment, as well as fear of chronic medication and its side effects [43]. Studies specifically assessing depression and anxiety suggest that children with EoE exhibit higher rates of both when compared to healthy control populations [41,43]. Evidence also highlights abnormal behavioral adjustment and poor emotional development as children get older, suggesting the importance of developmental considerations [23,42,43].

## 4. Conclusions

A progressive step-up empiric elimination diet in EoE is recommended for dietary therapy in EoE. Restrictions over a 4-FED should be carefully discussed with patients and parents and counseled by nutritionists. Malnourishment is not common in EoE patients and is likely related to concomitant IgE- and non-IgE-mediated comorbidities. These latter patients are not good candidates for dietary therapy. Avoidant/restrictive food intake disorder in pediatric EoE patients on highly restrictive diets has been lately described and may warrant specific psychological support. The psychosocial and emotional domains in the QoL of adult and child EoE patients may be impaired, more specifically disease and choking anxiety, psychosocial implications of dietary restrictions, and higher rates of depression.

## Figures and Tables

**Table 1:** Available empiric elimination diets in pediatric and adult EoE patients.

	Efficacy (<15 eos/HPF)		Food Triggers Identified		Potential Drawbacks
	Children	Adults	Children	Adults	
**Cow’s milk** **elimination diet** [8,10,11]	**51%**	**25–27%**	-	-	Methodological issues with all available studies in children (concomitant PPI therapy, selection bias) Indirect data from patients responders to a 2- and 4-FED
**2-FED** (milk and wheat) [10]	**44%**	**40%**	**68% one food trigger** Milk 52% Wheat 15% **28% two food triggers**		Single study requiring external validation. Egg might be more common than wheat as a food trigger in other settings All responders had 1 or 2 food triggers (best candidates for maintenance therapy)
**4-FED** (milk, wheat, egg and soy/legumes) [8,9]	**60%**	**46%**	**1 food 64%** **2 foods 20%** Milk 84% Wheat 28% Egg 8%	**1 food 45%** **2 foods 45%** **Milk 50%** Wheat 31% Egg 22%	Legumes beyond soy are more common as food triggers in Mediterranean countries. 80–90% of patients were found to have 1 or 2 food triggers (best candidates for maintenance therapy)
**6-FED** (milk, wheat, egg, soy/legumes, nuts, fish/seafood) [4,5,6]	**73%**	**71%**	**US** [4] Milk 74% Wheat 26% Egg 17%	**US** [5] Wheat 60% Milk 50% **Spain** [6] Milk 62% Wheat 29% Egg 26% Legumes 24%	Highly restrictive. Impairment of quality of life, psychosocial limitations. After stepping up from a 2- and 4-FED, responders to a 6-FED showed 3 or more causative foods (poor candidates for maintenance therapy)

**Table 2:** Potential reasons leading to failure to thrive/malnutrition in EoE patients.

**Concomitant mucosal gastrointestinal eosinophilic disorders (EGIDs), resulting in malabsorption** [26,27,28,29]
**Concomitant IgE-mediated food allergies** [28]
**Feeding difficulties due to symptoms** (regurgitation in toddlers, vomiting and reflux-like symptoms (children <10 years), food impaction/dysphagia in older children and adults) [1]
**Abnormal feeding behavior** (children: food refusal, low volume intake, slow pace, picky eating; adults: avoidance of solid foods, prolonged meals, drinking abundant liquids) [1,30,31]
**Avoidant/restrictive food intake disorder** (ARFID) [32,33]
**Concomitant comorbidities unrelated to EoE, EGIDs, and IgE-mediated food allergies** [23,30]
**Highly restrictive diets** (usually a combination of empirical diets plus IgE-mediated food allergies)
**Diagnostic delay in early studies** (some untreated patients may progress to severe fibrostricturing disease) [1]

**Table 3:** Clinical presentation of EoE with subsequent features of maladaptive feeding by age [1,30,31].

	<3 Years	4–10 Years	10–14 Years	Adolescents and Adults
**Symptoms**	Vomiting, irritability, pain	Abdominal pain, vomiting, regurgitation, heartburn	Dysphagia Heartburn	Food impaction Dysphagia
**Feeding dysfunction**	Low volume intake, food refusal, delayed oral feeding skills, grazing behavior	Food refusal, poor appetite, “picky eating”, trouble with inclusion of new foods to the diet, preference for softer foods and liquids, slow pace of eating	Slow eating pace, low food variety, preference for softer foods and liquids, anxiety during meals	Drinking abundant liquids to minimize dysphagia, avoidance of specific solid foods, slow pace of eating, fear and anxiety at mealtimes

**Table 4:** Diagnostic criteria for avoidant/restrictive food intake disorder (ARFID) [32,33].

**Feeding or eating disturbance as manifested by sustained failure to meet** **adequate nutritional and/or energy needs, associated with one of the following:** Significant nutritional deficiency Significant weight loss Dependence on enteral feeding or oral nutritional supplements Marked interference with psychosocial functioning **The eating disturbance is not attributable to a concurrent medical condition** **or not better explained by another mental disorder** **The disturbance is not better explained by culturally sanctioned practice or** **lack of available food** **There is no evidence of a disturbance in the way one’s body weight or shape is experienced.**

## Data Availability

Not applicable.

## Abbreviations

ARFID: avoidant/restrictive food intake disorder; BMI: body mass index; EGIDs: eosinophilic gastrointestinal disorders; EoE: eosinophilic esophagitis; QoL: quality of life; 2-FED: two-food elimination diet; 4-FED: four-food elimination diet; 6-FED: six-food elimination diet.
